# Supplementary material for: Rapid prediction of crucial hotspot interactions for icosahedral viral capsid self-assembly by energy landscape atlasing validated by mutagenesis
Source: PLoS Comput Biol. 2020 Oct 20;16(10):e1008357. doi: 10.1371/journal.pcbi.1008357 (PMC7598928; doi:10.1371/journal.pcbi.1008357)
Supplement: Attachment — Submitted filename: Response.pdf [file pcbi.1008357.s001.pdf]

# Reviewer 1

In the manuscript "Rapid prediction of crucial hotspot interactions for icosahedral viral capsid self-assembly by energy landscape atlasing validated by mutagenesis", Wu et al introduce a computational method to predict if mutations in capsid proteins can disrupt viral capsid assembly. There are currently no computational methods to assess this efficiently, and the research is significant. However, there are several major issues in the manuscript that challenged a rigorous scientific assessment of the research. The computational and experimental methods are incomplete, the comparison with experimental results is unclear, and the methodology introduces strong approximations that are not discussed with rigor. Below I listed my specific comments regarding these major issues as well as a number of minor issues.

Major issues:

1) The authors claim that the computational approach is validated experimentally (see, e.g., lines 119–121), but this comparison is unclear in the manuscript. I have a number of questions regarding this issue: Were the experiments carried in this research work, or were they published previously?

The AAV2 mutagenesis experiments were carried out expressly for the purpose of this study, subsequent to the prediction. The other in-vitro mutagenesis results have been collected from multiple papers, also subsequent to the prediction. These papers were previously listed in the results section. We have now made it clear everywhere including the abstract and introduction. And we have created an entire new table (Table 1) with this information and added a new figure (Figure 9) showing all the three VP monomers (AAV, MVM, BMV) highlighting the residues used in this study.

Section 3.1 Experimental setup provides limited information about the experiments (lines 437–448).

What where the mutations done for each virus and what were the assembly results of these experiments?

We have created an entire new table (Table 1) with this information.

Figures 10 and 11 provide the "validation" of the computational method and experiments, but it was not clear what was the prediction from the computational method, what was the experimental observations, and how they were compared.

The sub-figures in figure 10 (currently Figure 11) show a correlation between our first two-scale prediction and experimental observation (commonly used in 'support vector machine' learning) as follows.

In sub-figures in figure 10 (currently Figure 11), the points represent candidate hotspot residues. The coordinate values at which they have been placed, are our computational cruciality predictions.

The blue and red coloring of the points indicate residues found through mutagenesis to disrupt and not disrupt assembly respectively. The green circles are residues on which no mutagenesis was performed.

The figures show that the predicted cruciality values of the residues that were later shown to disrupt assembly are linearly separated from the predicted cruciality values of the residues that do not disrupt assembly. I.e., the prediction convex hull formed by assembly-disrupting residues does not significantly intersect the convex hull formed by the non-assembly-disrupting residues.

Conversely, if there were poor correlation between prediction and experiment, such a linear separation (or separation of convex hulls) would not be possible.

In figure 11 (currently Figure 12), the correlation between our second two-scale prediction and experiment is illustrated by the correlation between the prediction ranking of cruciality (top to bottom) and the experimentally shown extent of assembly disruption (color blue to red).

More precisely, the residues listed on the top of the table are computationally predicted through our method to be more crucial, and the ones listed lower are predicted as less crucial. On the other hand, residues experimentally shown to strongly disrupt assembly are blue and those that do not disrupt are red, with partial disruption indicated by the spectrum of colors in between.

These have now been made clear in the manuscript.

2) Some parts of the computational method are described in great detail, but several elements that are key to judge the application of this method to the three viruses studied are missing:

How were the 3D atomic models of the assembled viruses used to define the discretized Lennard-Jones potentials?

In the manuscript, our choice of the Lennard-Jones potential is explained as follows

"The potential energy includes the hard-sphere potential between all atom pairs (one from each participating VP monomer or dimer) with the Van der Waals radius set to 1.2 Å. We used Lennard-Jones pair potentials, setting the energy difference of Equation (4) to $E_h - E_l = 0.997 kJ/Mol$ [92], and the weight in Equation (5) to $w(N_a) \approx 1.5^{N_a}$ . An implicit solvent was assumed."

How sensitive are the results of the computational method to variations to the Lennard-Jones pair potential energy difference?

How sensitive are the results of the computational method to variations in the criteria of assignment of the Lennard-Jones discrete potential to atom pairs?

This is a good question which we have now addressed. As you point out, the two aspects of the Lennard-Jones potential that could possibly affect the results are (a) the energy difference and (b) the width we set for the discretized Lennard-Jones well. We observe that our predictions are not sensitive to the energy difference, whereas the width of the discretized Lennard-Jones is completely determined from the given capsid assembly configuration, which is assumed to be a local energy minimum. To elaborate further: our prediction method relies significantly more on the width of the discretized Lennard-Jones well rather than the actual energy difference $E_h - E_l$. The number of atom-pairs within the well essentially determines the quantity $N_a$ which is the deciding quantity in the computation of the normalized partition function affecting the cruciality prediction. However, since we know that the given capsid assembly configuration is feasible, the minimum pairwise distance (which in all cases turned out to be Van der Waals) forces the lower bound of the width of the discretized Lennard-Jones well. On the other hand, since the given capsid assembly configuration is a local energy minimum with (at least) 6 atom-pairs within the Lennard-Jones well, we can measure a natural upper bound value for the width of the discretized Lennard-Jones well (a larger value would permit a energy-neutral motion within the basin to a configuration with more atom-pairs in the Lennard-Jones well, and hence lower-energy, contradicting the minimality of the given capsid assembly configuration.)

3) The methodology assumes a quasi-equilibrium pathway of assembly, where the partial capsids can be mapped onto the final capsid configuration. This is a strong approximation that disregard thermodynamic and kinetic mechanisms established previously. The limitations of the model should be put in context with the current knowledge of assembly pathways (see for example, Reguera et al Soft Matter 2019, Ning et al Nature Communications 2016, Hagan and Elrad Bioph J 2010, and the references within).

We thank the reviewer for directing us to these additional references. We have now incorporated them in our related work. We acknowledge that kinetics have clearly been shown to affect many aspects of assembly. In the related work section, we have reviewed methods that separately take kinetics into account and have clearly stated that "... While our method does not *separately* model kinetics, it combines interface-scale and capsid-scale analyses of free-energy and configurational/combinatorial entropy, which could affect kinetics" Our predictions do strongly rely on thermodynamics, specifically free energy. We believe that our results show that geometry and consequently configurational entropy may already be determinative in answering the narrow question of which inter-atomic interactions are more or less crucial to assembly, at least for the viral capsids studied here.

Minor issues

1) Several terms like cruciality, subassembly, or atlasing are used early in the manuscript without having been properly defined. Some of the terms are defined later on in the manuscript, but it takes several pages. The authors should provide definitions for these terms early on in the manuscript.

We have defined cruciality in the abstract and introduction as the "importance (cruciality) of an interface for assembly ..."

We have now defined subassembly in the introduction as follows. 'subassemblies are intermediate oligomeric structures of the capsid, assembled at interfaces ...'

atlasing has been defined in the introduction in the paragraph starting line 74.

2) The title of the manuscript claims that is a "rapid prediction" but this claim is based on a vague sentence referring to the computational time needed on a "laptop". The specs of this laptop should be provided (CPU, RAM, number of processors...) so the community can properly judge this computational time.

The computational experiments were carried out on a laptop with Intel(R) Core(TM) i5-2500K @ 3.2 Ghz CPU and 8GB of RAM. We have clarified this in the text now.

How long did they take?

The atlasing took no more than 1.5 hours per interface. We have clarified this in the text now.

3) The authors claim that “Icosahedral capsid self-assembly is poorly understood process” (lines 13 and 14). This is misleading. There are various principles (nucleation process, weak interactions, etc.) that are well established (see references given above). This includes the assumptions used in the computational method presented here. The authors should acknowledge this properly and clarify the aspects that remain open (like the multi-scale aspect).

We have changed the sentence to the following

“While several aspects of icosahedral capsid self-assembly have been studied in detail, its multi-scale aspect still remains poorly understood.”

4) Figure 1a is unclear. In a T=1 each monomer is involved equivalently in each icosahedral axis of symmetry. All proteins are equivalent in such capsid. Do the colors associated to each axis reflect domains in the monomers?

The non-dominant colors (colors other than red) have been used to merely delineate the different interfaces in a T=1 virus. To this end, we have highlighted using colors (green, blue, and yellow), one interface of each type (5-fold, 3-fold, and 2-fold).

5) Review grammar throughout the text, for example (lines 13-14, lines 44-45, . . . )

We have carefully proof read the entire document for grammatical mistakes now.

# Reviewer 2

The manuscript deals with computational modeling of the assembly of icosahedral viruses. Thereby, interface-scale and capsid-scale modeling approaches are combined. The approach uses the EASAL software of the same group. Overall, the is well written and provides detailed explanations of the complex methods.

The only point is that the validation of the prediction with AAV2, MVM and BMV mutagenesis results. The statement of strong correlation in section 3.3 is not really supported by Figure 11. The authors should elaborate this point more detailed or discuss it more cautiously.

We believe that the results for AAV2 and MVM do show a strong correlation (we found the extent of correlation surprising!) We do agree with the reviewer about the paucity of results of BMV. We have now included the following text to further explain the results of BMV.

While the results for BMV are sparse and hence do not exhibit the strong correlation seen in the results of the other two viruses, the results still correctly predict the cruciality for most of the residues for which experimental results are available. We believe the lack of the strong correlation to be an artifact of the small amount of mutagenesis data available for training the model.
