# Supplementary material for: Rapid prediction of crucial hotspot interactions for icosahedral viral capsid self-assembly by energy landscape atlasing validated by mutagenesis
Source: PLoS Comput Biol. 2020 Oct 20;16(10):e1008357. doi: 10.1371/journal.pcbi.1008357 (PMC7598928; doi:10.1371/journal.pcbi.1008357)
Supplement: Attachment — Submitted filename: Response.pdf [file pcbi.1008357.s002.pdf]

Reviewer 1

Regarding the weaker correlation for the BMV case, I would like the authors to consider the following observation: AAV2 and MVM both form and empty capsid followed by the packing of the ssDNA genome. BMV instead co-assembles the capsid with the ssRNA genome, which plays an essential role in coordinating the assembly (Comas-Garcia, Viruses, 11:253, 2019). Empty BMV capsids can assemble in vitro, but the BMV wild-type has selected capsid proteins to co-assemble with the genome. Since the algorithm relies exclusively on information about capsid proteins, the approach seems better suited for viruses that form procapsids rather than viruses that co-assemble with the genome. The information about potential hot spots taking into account co-assembly information is missing in the current approach and might explain the issues the BMV results.

We have discussed the reviewer's insightful comment about BMV in the discussion section of the manuscript, and acknowledged it in the acknowledgments section. In addition, we have moved into the main paper a complete ranking table that was previously in the supporting information. This is the full ranking of our two-scale model - including residues for which in vitro results are unavailable - to demonstrate the paucity of data for BMV and to explain the less conclusive correlation between computational prediction and in-vitro experimental data compared to the other two viruses.

Other changes

In addition to including the above explanation, we have made the following changes to the paper.

1. We have removed two authors of the manuscript for the following reason. The manuscript uses site-directed mutagenesis biophysical assay results from the literature for 3 viruses - AAV2, MVM and BMV - to validate our computational predictions. Of these, the AAV2 experiments had been performed by Dr. Agbandje-Mckenna and Dr. Bennet contemporaneously with the development of our computational model and prediction. Hence, previously, we had not only cited their result as an unpublished manuscript (Dr. Bennet's PhD thesis), but also included them as coauthors.

Dr. Agbandje-Mckenna and Dr. Bennet feel that as the manuscript is entirely about computational predictions with almost no details of the biophysical assays, and we were citing their results anyway, it was sufficient to acknowledge their contributions in the acknowledgments section and not include them as coauthors. Consequently we have changed the few sentences referring to the AAV2 experiments, removed them as coauthors, and listed their contributions in the acknowledgments section.

2. In accordance with the above point, we have now removed the short paragraph description of the biophysical assays from Section 3.1 Experimental Setup.

3. We have edited Figure 2 to clearly say "validation by site directed mutagenesis biophysical assay results".

4. We have reordered the entries in Table 1 and sorted them by the residue id to clarify exactly what was used for validation (the previous ordering incorporated our prediction rankings of Figures 12 and 13.)

5. We have improved Figure 9 so that the candidate residues are visible.
