# Supplementary material for: Rapid prediction of crucial hotspot interactions for icosahedral viral capsid self-assembly by energy landscape atlasing validated by mutagenesis
Source: PLoS Comput Biol. 2020 Oct 20;16(10):e1008357. doi: 10.1371/journal.pcbi.1008357 (PMC7598928; doi:10.1371/journal.pcbi.1008357)
Supplement: Attachment — Submitted filename: Response2.pdf [file pcbi.1008357.s003.pdf]

Dear Editors,

Per the Sep 19, 2021 email from Dr. Susan Ellison, we are uploading

- clean manuscript without figures, and without symbols such as (R) (TM) etc.
- manuscript tracking the abovementioned changes, since no other changes have been requested after we submitted the Previous Revision 2
- separate figure files including Figure 10
- we note that the supplemental information section of the paper contains links to a website where data and code are provided in a reader-friendly format
- cover letter indicating the above
- there was no review, hence we have no response to reviewers, but since that item is mandatory, we have just uploaded the coverletter.

Thanks,

Meera Sitharam
